# The impact of different Zinc (Zn) levels on growth and nutrient uptake of Basil (*Ocimum basilicum* L.) grown under salinity stress

**DOI:** 10.1371/journal.pone.0246493

**Published:** 2021-02-02

**Authors:** Inci Tolay

**Affiliations:** Department of Soil Science and Plant Nutrition, Akdeniz University, Faculty of Agriculture, Antalya, Turkey; Harran University, TURKEY

## Abstract

Salinity is among the most important abiotic stresses, which negatively affect growth, nutrient uptake and yield of crop plants. Application of different micronutrients, particularly zinc (Zn) have the potential to ameliorate the negative impacts of salinity stress. However, the role of Zn in improving salinity tolerance of basil (*Ocimum basilicum* L.) is poorly understood. This study evaluated the impact of different Zn levels (0, 5 and 10 mg kg^-1^) on growth and nutrient acquisition traits of basil under different salinity levels (0, 0.5, 1.0 and 1.5% NaCl). Data relating to biomass production, chlorophyll index, sodium (Na), potassium (K) uptake, K/Na ratio, Zn, copper (Cu), manganese (Mn) and iron (Fe) uptake were recorded. Increasing salinity level reduced biomass production, chlorophyll index and nutrient uptake traits (except for Na and Fe accumulation) of basil. Zinc application (10 mg kg^-1^) improved biomass production, chlorophyll index and nutrient acquisition traits under normal as well as saline conditions. The reduction in chlorophyll index and biomass production was higher under 0 and 5 mg kg^-1^ than 10 mg kg^-1^ Zn application. The K concentration decreased under increasing salinity; however, Zn application improved K uptake under normal as well as saline conditions. Different growth and nutrient acquisition traits had negative correlations with Na accumulation; however, no positive correlation was recorded among growth and nutrient uptake traits. The results revealed that Zn application could improve the salinity tolerance of basil. However, actual biochemical and genetic mechanisms involved in Zn-induced salinity tolerance warrant further investigation.

## Introduction

Salinity is an important constraint for crop production in many geographic regions of the world, and frequently occurs in irrigated lands of arid and semi-arid regions [1]. Irrigation water containing trace amounts of sodium chloride (NaCl) increases salt levels in arable soils [2, 3]. Globally, salinity affects 831 million hectares of land [4], and the saline area is increasing with each passing day [5]. Salinity excludes 1.5 million hectares of productive lands from agricultural production each year [3]. Salinity is of important concern for salt sensitive crops grown in arid zones [6, 7]. Soil and water salinity are major constraints in global food production, particularly in semi-arid and arid regions [8]. Saline groundwater is commonly used to fulfill moisture requirements of crops sown in areas with limited water resources [9–12]. Nonetheless, recycling of wastewater and its use for irrigation are also gaining popularity [13, 14].

Salinity is among the most important abiotic stresses, which limit plant production; thus, studied for many years. Salinity can directly damage plants, or inhibit plant growth depending on salinity-tolerance level of plants and salt concentration in the environment [3, 14]. Salinity induces chlorophyll and membrane breakdown (chlorosis and necrosis) starting from old leaves [15, 16]. Salinity causes toxicity and mineral nutritional disorders in plants, ultimately resulting in disturbed metabolism [15]. Thus, salinity causes both qualitative and qualitative yield losses by limiting plant growth [17]. The growth-limiting factors for plant growing under saline environments can be categorized in 3 different groups [15], which are water stress, Na^+^ and Cl^-^ toxicity and associated nutrient uptake, ion toxicities and deficiency of K^+^ and Ca^++^.

Reclamation of saline soils through leaching soil profile is frequently recommended in the literature to eliminate negative consequences on plant growth [18, 19]. However, this approach is time-consuming and costly. In addition, salinity mostly occurs in arid and semi-arid regions where water-based solutions are not practical. While salinity is a common problem of arid and semi-arid regions, zinc (Zn) deficiency also impairs plant production in the same regions. Although yield and quality of plants are negatively affected by salinity around the world [17, 20], Zn deficiency often occurs in calcareous, saline and sodic soils with high pH values [21, 22]. In addition to adverse impacts of Zn deficiency on yield and quality of plants, it is also a serious problem for human nutrition.

Zinc reduces excessive Na uptake under saline environments through affecting structural integrity and permeability of stem cell membrane [23]. Zinc nutrition is effective in decreasing Na accumulation and improving K/Na ratio in plants under salinity [1, 24]. Therefore, cell membranes show high permeability or leakage of some compounds from the roots under Zn deficiency [25]. Zinc deficiency can lead to accumulation of toxic ions such as Na and Cl. Therefore, combined effects of salinity and Zn-deficiency on plant growth are important and need investigation.

Basil (*Ocimum basilicum* L.), a member of *Lamiaceae* is an annual, herbaceous plant of Mediterranean regions. The basil is rich in antioxidant and phenolic compounds, such as rosmarinic acid and other cafeic acid derivatives, and is regarded as a source of aromatic compounds [26]. The plant is grown as a medicinal and spice plant in many countries of the world [27, 28]. The large consumption of basil as a food ingredient makes it a possible candidate of bio-fortification.

Although impacts of salinity and Zn have been investigated on many plants, there are almost no studies carried out on basil. Therefore, this study determined the growth, biomass production and nutrient uptake response of basil under different salinity and Zn application levels. It was hypothesized that increasing salinity level will suppress the growth and nutrient uptake, whereas increasing Zn levels will ameliorate the negative consequences of salinity on basil.

## Materials and methods

### Experimental site

The study was conducted on basil population grown in Aegean Region. A calcareous and Zn-deficient soil having DTPA extractable Zn level of 0.20 mg kg^-1^ was used in the study. The pH of the experimental soil and total salts were analyzed following Jackson [29]. The method of Bouyoucos [30] was followed to determine soil texture. Organic matter was analyzed according to Walkley and Black [31]. Total phosphorus (P) and potassium (K) were analyzed by following Olsen [32] and Carson [33], respectively. Iron (Fe), zinc (Zn), manganese (Mn) and copper (Cu) were analyzed according to Lindsay and Norvell [34]. The experimental soil was slightly alkaline (pH 8.02), non-saline (0.24 mmhos cm^-1^), clay-loam, low in organic matter (1.1%), moderately calcareous (10.2%), low in available P (4.8 mg kg^-1^), sufficient in available K (149 mg kg^-1^), Zn-deficit (0.2 mg kg^-1^), medium in Fe (0.85 mg kg^-1^), low in Mn (2.74 mg kg^-1^) and sufficient in Cu content (0.46 mg kg^-1^).

### Experimental treatments

Four salinity levels (i.e., 0, 0.5, 1 and 1.5% NaCl) were used in the experiment. Similarly, three Zn application levels [i.e., Zn_0_ = 0 mg kg^-1^, Zn_5_ = 5 mg kg^-1^ and Zn_10_ = 10 mg kg^-1^ Zn (in the form of Zn SO_4_.7H_2_O)] were included in the study. All treatments had three replications. The experiments were performed in greenhouse of Çukurova University, Faculty of Agriculture, Department of Soil Science and Plant Nutrition. Basic nutrients, i.e., 200 mg kg^-1^ N in the form NH_4_SO_4_, 100 mg kg^-1^ P and 125 mg kg^-1^ K in the form of KH_2_PO_4_ and 2.5 mg kg^-1^ Fe in the form of Fe-EDTA were applied. The pots were filled with 1.65 kg of soil and 20 seeds were planted in each pot. After seed germination, plants were reduced to 10 per pot. Salinity was imposed through irrigation water three times with an interval of two days [35] and 0, 0.5, 1 and 1.5% (w/v) NaCl solutions were applied 53 days after planting. The pots of salinity-free treatment were maintained at field capacity by irrigating with distilled water. Experiments were conducted according to factorial design where salinity was considered as main factor, while Zn application levels were regarded as sub-factor. There were no specific permits required for the experiments since no endangered/protected species were involved in the study.

### Data collection

The SPAD values were determined at the end of experiment. Aboveground parts were harvested on the 72nd day depending on Zn deficiency symptoms observed in control treatment. The harvested plants were dried at 65°C for 48 hours to determine biomass production. The dried plants were weighed and a pre-weighed quantity was burnt in H_2_O_2_-HNO_3_ acid mixture in a closed system (Milestone 1200 Mega) microwave oven for Zn analysis. The Zn, K and Na concentrations in the obtained filtrate were measured in an Inductively Coupled Plasma-Atomic Emission Spectrometry (ICP-AES) device.

### Statistical analysis

The collected data were tested for normality and homogeneity of variance first, which indicated a normal distribution. Two-way analysis of variance (ANOVA) was used to infer significance in the data. Least significant difference test at 5% probability level was used as a post-hoc test to separate the means. All statistical analyses were performed on SPSS version 20.0.

## Results

Salinity and zinc (Zn) levels and their interaction significantly altered biomass production and chlorophyll index (Table 1).

**Table 1:** Analysis of variance for biomass production, chlorophyll index, nutrient acquisition traits and K/Na ratio of Basil (*Ocimum basilicum* L.) grown under different NaCl salinity and zinc (Zn) levels.

Source	DF	Sum of squares	Mean squares	F value	P value
**Biomass production**					
Salinity levels (S)	3	0.04	0.01	86.91	< 0.0001*
Zn levels (Zn)	2	0.01	0.01	43.93	< 0.0001*
S × Zn	6	0.01	0.00	6.28	0.0005*
**Chlorophyll index**					
Salinity levels (S)	3	2545.04	848.35	233.87	< 0.0001*
Zn levels (Zn)	2	389.98	194.99	53.75	< 0.0001*
S × Zn	6	332.56	55.43	15.28	< 0.0001*
**Na accumulation**					
Salinity levels (S)	3	192.13	64.04	1822.43	< 0.0001*
Zn levels (Zn)	2	3.81	1.91	54.27	< 0.0001*
S × Zn	6	17.23	2.87	81.71	< 0.0001*
**K accumulation**					
Salinity levels (S)	3	15.00	5.00	108.44	< 0.0001*
Zn levels (Zn)	2	0.57	0.28	6.15	0.0070^NS^
S × Zn	6	0.57	0.09	2.04	0.0988^NS^
**K/Na ratio**					
Salinity levels (S)	3	24.59	8.20	617.74	< 0.0001*
Zn levels (Zn)	2	1.64	0.82	61.77	< 0.0001*
S × Zn	6	2.10	0.35	26.39	< 0.0001*
**Zn accumulation**					
Salinity levels (S)	3	92.92	30.97	4.27	0.001*
Zn levels (Zn)	2	19529.54	9764.77	1346.40	< 0.0001*
S × Zn	6	462.95	77.16	10.64	< 0.0001*
**Cu accumulation**					
Salinity levels (S)	3	2.08	0.69	4.83	0.0090*
Zn levels (Zn)	2	9.77	4.88	34.08	< 0.0001*
S × Zn	6	11.01	1.83	12.80	< 0.0001*
**Mn accumulation**					
Salinity levels (S)	3	4820.70	1606.90	75.74	< 0.0001*
Zn levels (Zn)	2	1197.66	598.83	28.23	< 0.0001*
S × Zn	6	1764.52	294.09	13.86	< 0.0001*
**Fe accumulation**					
Salinity levels (S)	3	68544.94	22848.31	547.18	< 0.0001*
Zn levels (Zn)	2	9818.04	4909.02	117.56	< 0.0001*
S × Zn	6	45133.57	7522.26	180.15	< 0.0001*

DF = degree of freedom

* = significant, NS = non-significant

**Fig 1:**
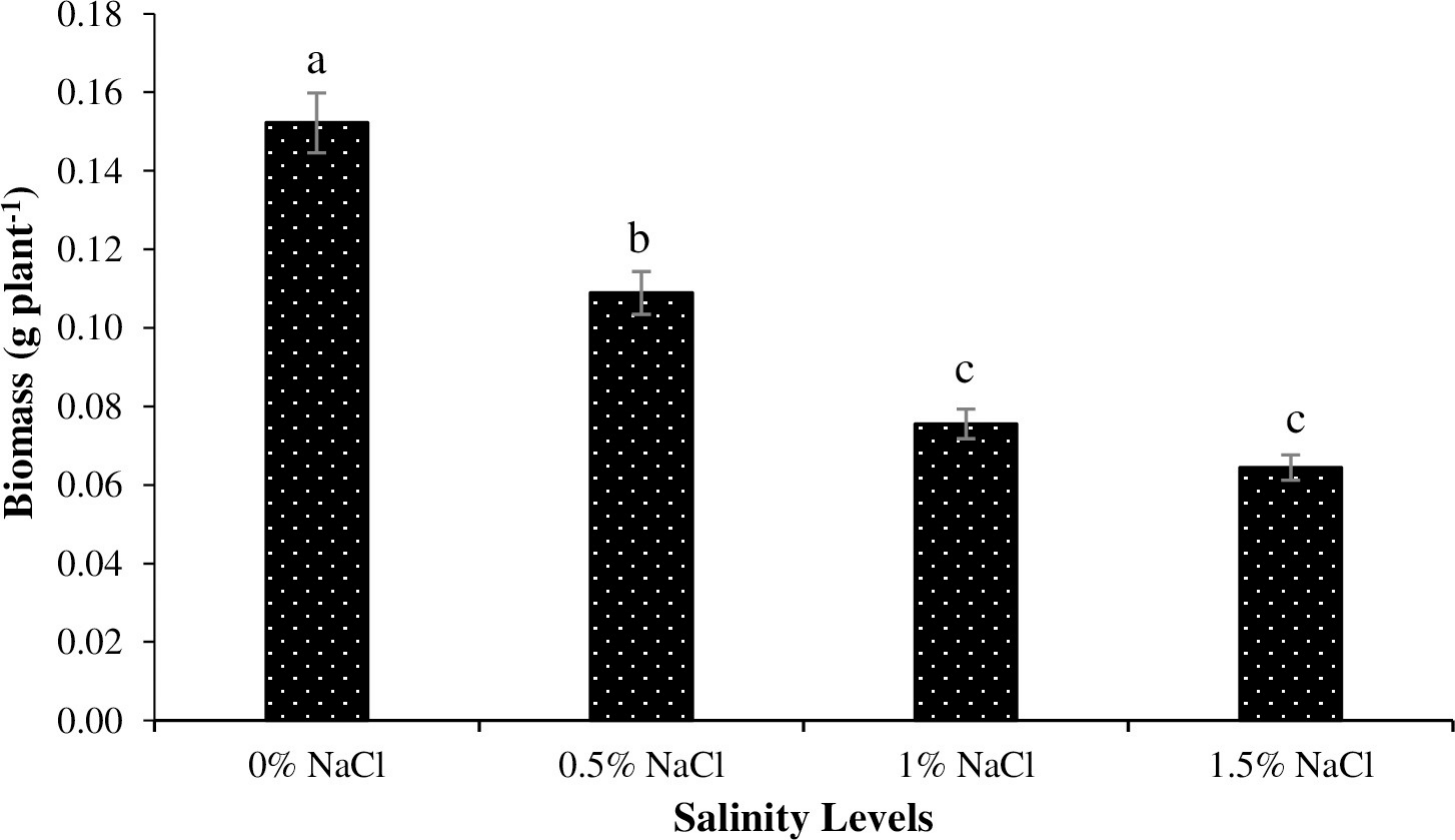
The influence of different NaCl salinity levels on dry biomass production of Basil (*Ocimum basilicum* L.). The vertical bars are means ± standard errors. Any two means having different letters are statistically different from each other (p < 0.05).

**Fig 2:**
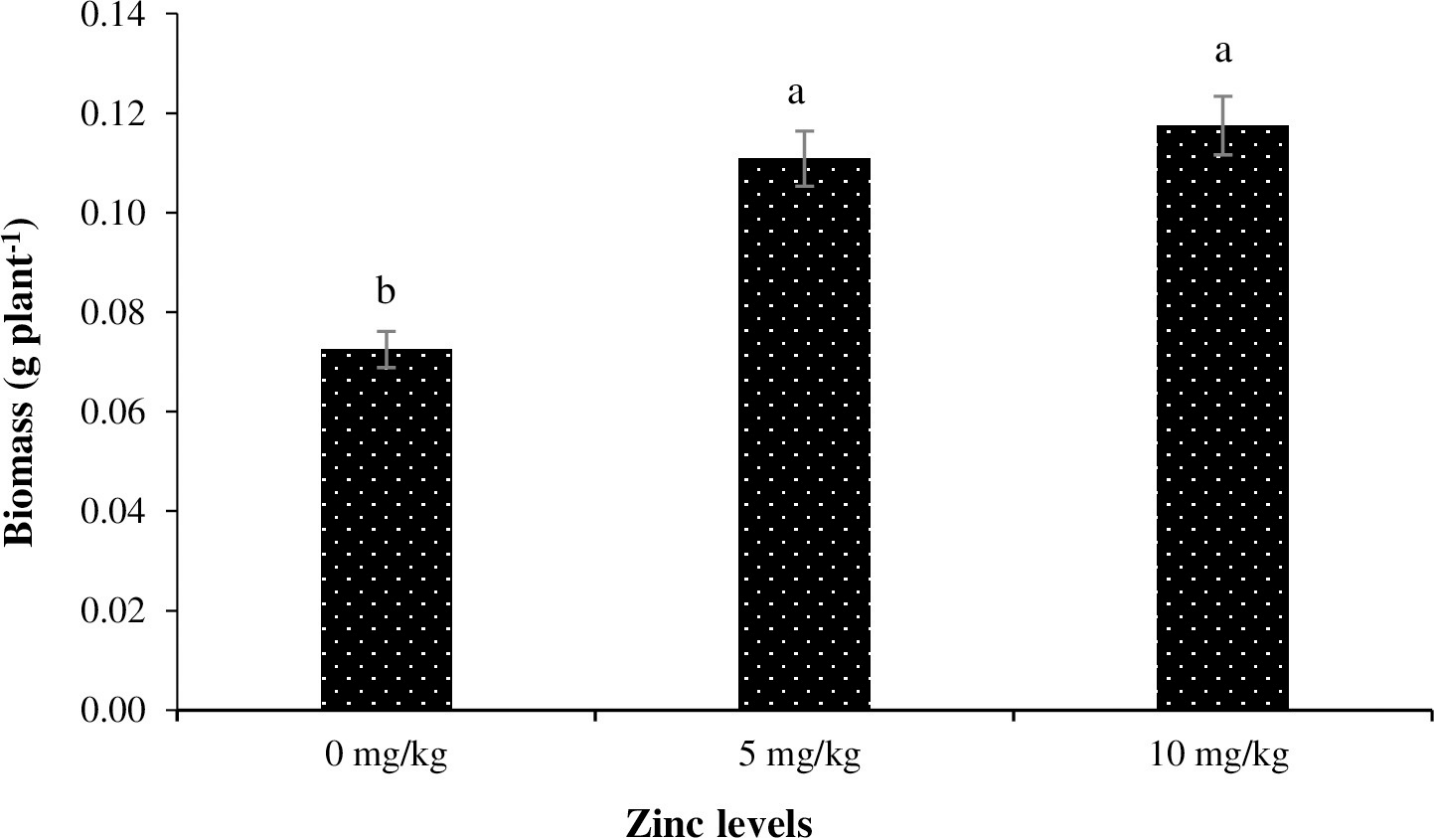
The influence of different Zinc (Zn) levels on dry biomass production of Basil (*Ocimum basilicum* L.). The vertical bars are means ± standard errors. Any two means having different letters are statistically different from each other (p < 0.05).

**Fig 3:**
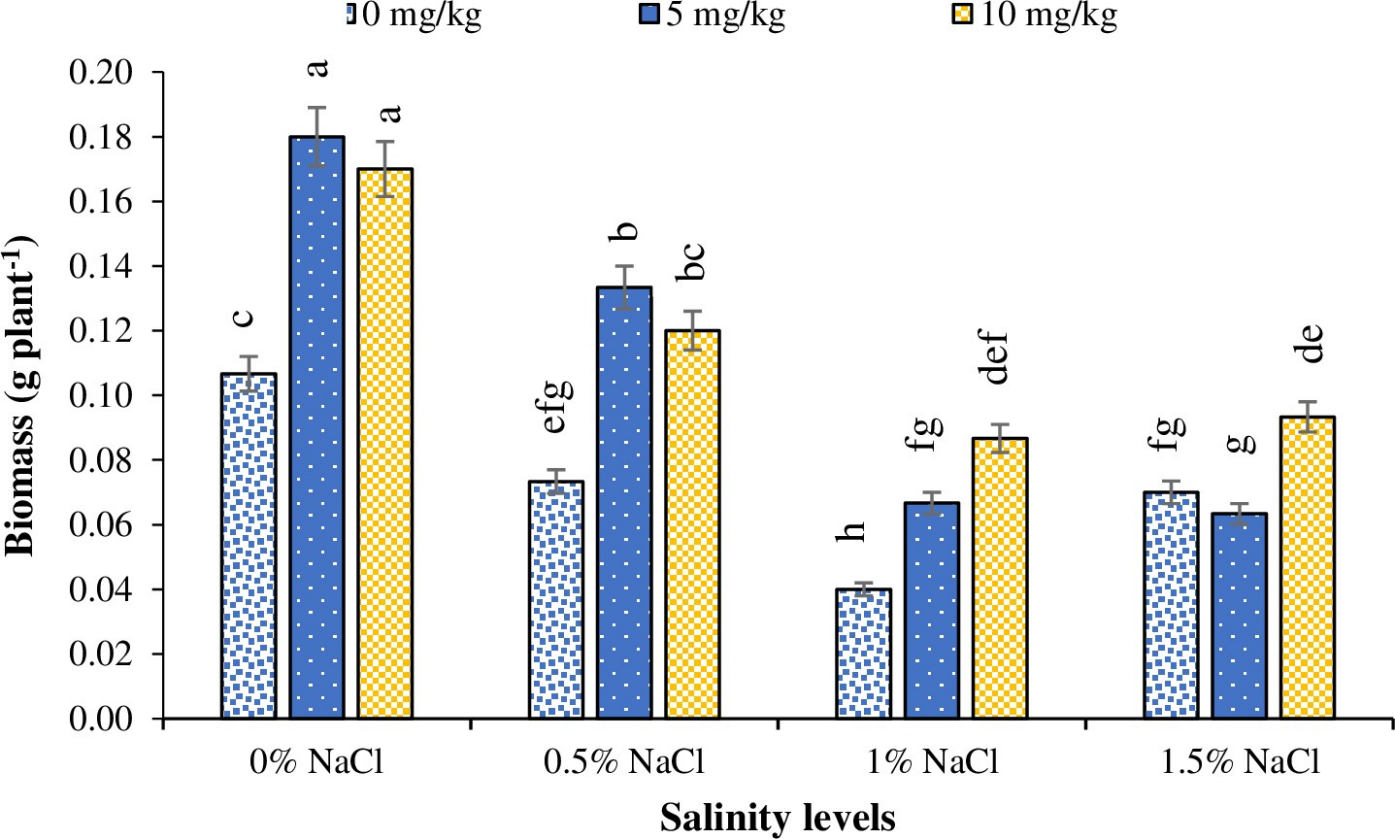
The influence of different zinc levels on dry biomass production of Basil (*Ocimum basilicum* L.) grown under different NaCl salinity levels. The vertical bars are means ± standard errors. Any two means having different letters are statistically different from each other (p < 0.05).

Plants grown under salinity-free environment produced the highest biomass, whereas those grown under 1.5% salinity level produced the lowest biomass (Fig 1). Similarly, the lowest biomass production was recorded for the plants grown under no Zn application, whereas plants grown under Zn_5_ and Zn_10_ levels produced the highest biomass (Fig 2). Regarding interaction among salinity and Zn levels, plants grown under 1% salinity and no Zn treatment had the lowest biomass production, whereas no salinity with Zn_5_ and Zn_10_ treatments resulted in the highest biomass production (Fig 3).

**Fig 4:**
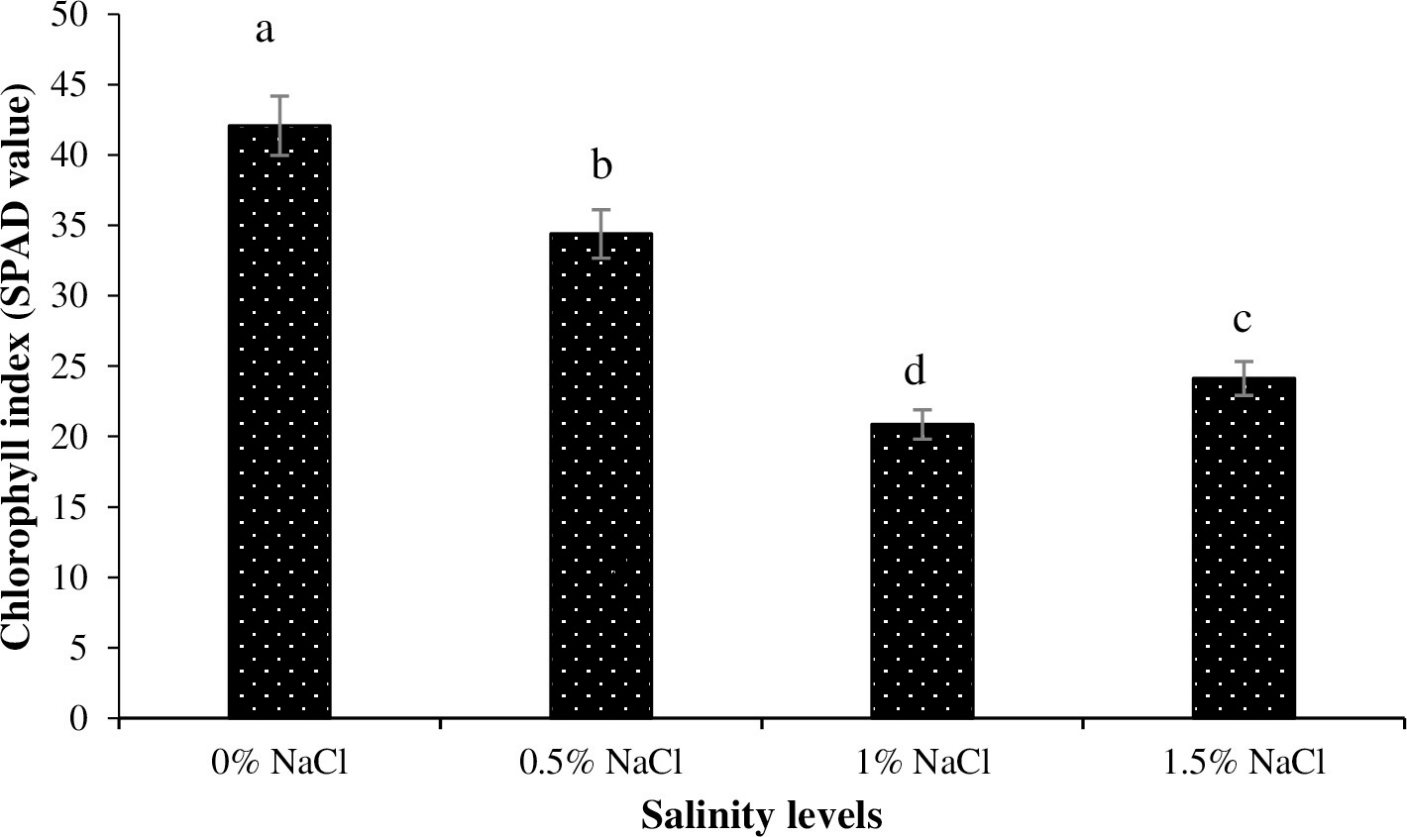
The influence of different NaCl salinity levels on chlorophyll index of Basil (*Ocimum basilicum* L.). The vertical bars are means ± standard errors. Any two means having different letters are statistically different from each other (p < 0.05).

**Fig 5:**
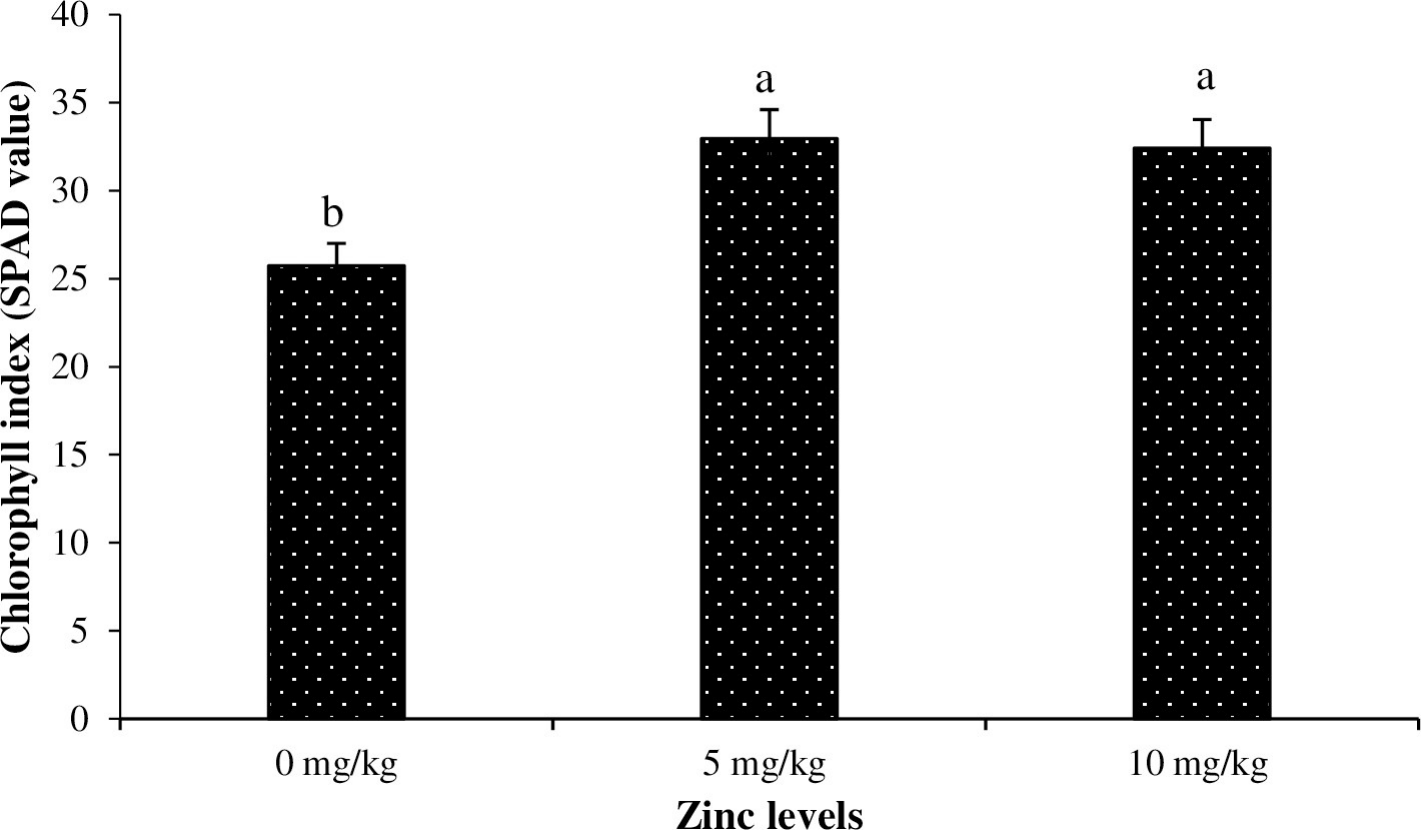
The influence of different Zinc (Zn) levels on chlorophyll index of Basil (*Ocimum basilicum* L.). The vertical bars are means ± standard errors. Any two means having different letters are statistically different from each other (p < 0.05).

**Fig 6:**
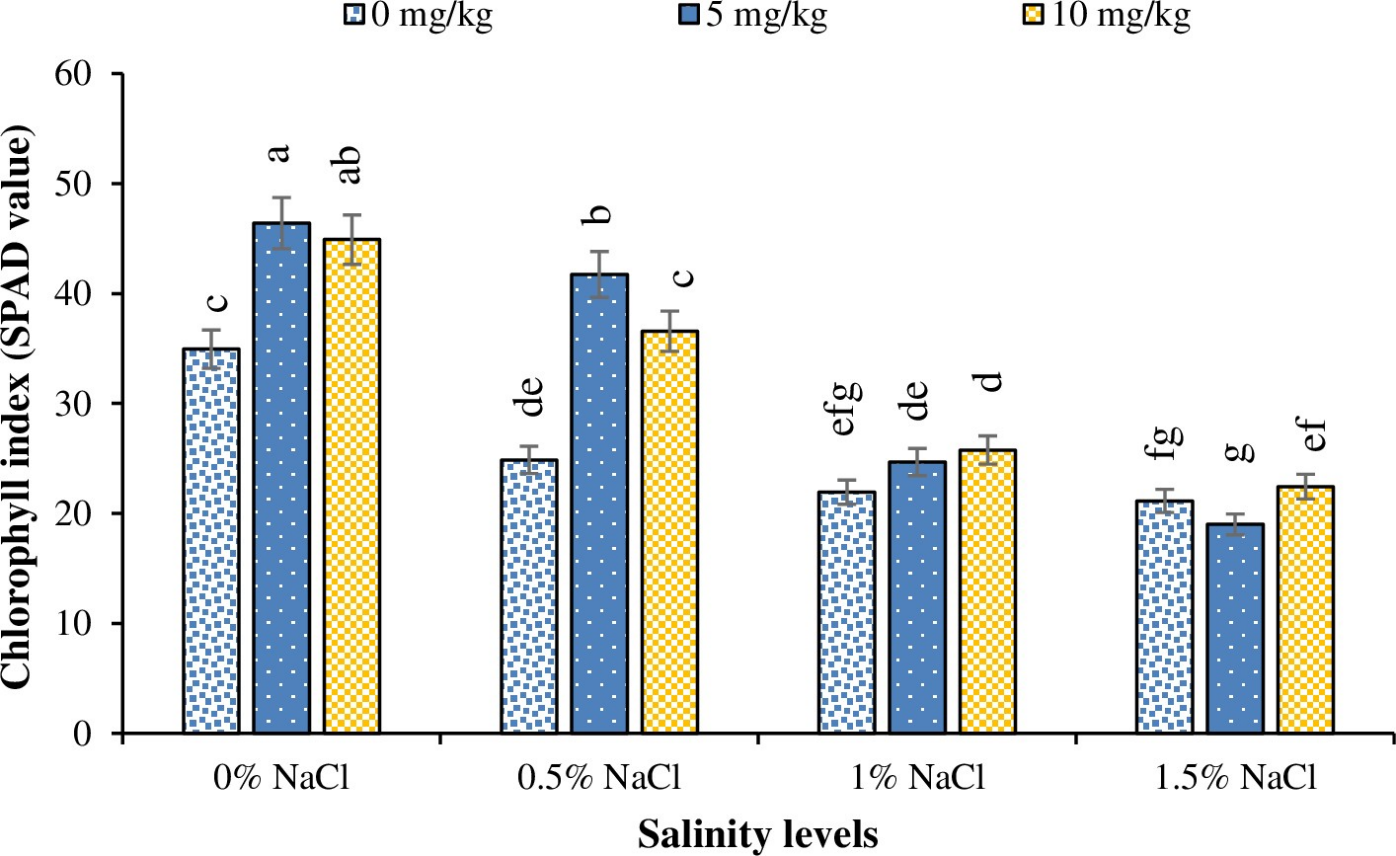
The influence of different Zinc (Zn) levels on chlorophyll index of Basil (*Ocimum basilicum* L.) grown under different NaCl salinity levels. The vertical bars are means ± standard errors. Any two means having different letters are statistically different from each other (p < 0.05).

The lowest chlorophyll index was noted for the plants grown under 1.5% salinity, whereas plants grown under no salinity had the highest chlorophyll index (Fig 4). Similarly, the lowest and the highest chlorophyll index was recorded for the plants grown under Zn_0_, Zn_5_ and Zn_10_ levels, respectively (Fig 5). Regarding interaction of salinity × Zn levels, plants grown under 1.5% salinity and Zn_5_ had the lowest chlorophyll index, whereas no salinity with Zn_5_ resulted in the highest chlorophyll index (Fig 6).

Individual and interactive effects of salinity and Zn levels significantly affected different nutrient acquisition traits such as sodium (Na), potassium (K), K/Na ratio, Zn, copper (Cu), manganese (Mn) and iron (Fe) uptake (Table 1). The highest and the lowest Na accumulation was recorded for plants grown under 1.5 and 0% salinity, respectively. Similarly, plants grown under Zn_5_ acquired the highest amount of Na, whereas 10 mg kg^-1^ Zn resulted in the lowest Na accumulation. Nonetheless, plants grown under no salinity and 10 mg kg^-1^ Zn accumulated the lowest amount of Na, whereas 1.5% salinity and 5 mg kg^-1^ Zn resulted in the highest Na accumulation (Table 2).

**Table 2:** The influence of different zinc (Zn) levels on nutrient acquisition traits and K/Na ratio of Basil (*Ocimum basilicum* L.) grown under different NaCl salinity levels.

Treatment	Na	K	K/Na	Zn	Cu	Mn	Fe
	(%)	(%)		(mg kg^-1^)	(mg kg^-1^)	(mg kg^-1^)	(mg kg^-1^)
**Salinity levels**							
**0%**	2.28 d	5.82 a	2.62 a	45.99 ab	9.29 ab	168.82 a	147.58 b
**0.5%**	3.69 c	4.37 b	1.35 b	47.36 a	9.40 a	167.57 a	88.54 d
**1%**	6.61 b	4.27 b	0.65 c	43.14 c	8.79 c	156.84 b	118.87 c
**1.5%**	8.12 a	4.38 b	0.54 c	44.29bc	9.00bc	139.94 c	206.82 a
**LSD 0.05**	**0.18**	**0.20**	**0.23**	**2.62**	**0.36**	**4.48**	**6.28**
**Zinc levels**							
**0 mg kg**^**-1**^	5.24 b	4.57 b	1.12 b	15.11 c	8.41 c	165.78 a	156.55 a
**5 mg kg**^**-1**^	5.54 a	4.87 a	1.15 b	48.63 b	9.64 a	157.35 b	147.06 b
**10 mg kg**^**-1**^	4.75 c	4.69 ab	1.59 a	71.85 a	9.31 b	151.75 c	117.75 c
**LSD 0.05**	**0.15**	**0.18**	**0.21**	**2.26**	**0.31**	**3.88**	**5.44**
**Salinity × zinc interaction**							
**S**_**1**_**Zn**_**1**_	2.27 h	5.45 b	2.40 b	12.37 f	7.87fg	186.10 a	104.03 f
**S**_**1**_**Zn**_**2**_	2.67 g	6.00 a	2.25 b	56.10 c	10.63 a	158.47 d	163.23 d
**S**_**1**_**Zn**_**3**_	1.89i	6.02 a	3.19 a	69.50 b	9.37bcd	161.90 cd	175.47 c
**S**_**2**_**Zn**_**1**_	5.17 e	4.38 cd	0.85 e	14.00ef	9.63 b	170.43 b	96.97fg
**S**_**2**_**Zn**_**2**_	3.74 f	4.42 cd	1.18 d	48.23 d	9.77 b	165.07bcd	88.57gh
**S**_**2**_**Zn**_**3**_	2.15 hi	4.30 d	2.02 c	79.83 a	8.80 de	167.20bc	80.10 h
**S**_**3**_**Zn**_**1**_	6.51 d	4.21 d	0.65 f	16.20ef	7.67 g	168.33bc	131.73 e
**S**_**3**_**Zn**_**2**_	6.76 cd	4.38 cd	0.65 f	45.07 d	9.20bcd	163.60bcd	138.03 e
**S**_**3**_**Zn**_**3**_	6.54 d	4.21 d	0.64 f	68.17 b	9.50bc	138.60 e	86.83gh
**S**_**4**_**Zn**_**1**_	6.98 c	4.21 d	0.60 f	17.87 e	8.47ef	138.27 e	293.47 a
**S**_**4**_**Zn**_**2**_	8.98 a	4.68 c	0.52 f	45.10 d	8.97cde	142.27 e	198.40 b
**S**_**4**_**Zn**_**3**_	8.41 b	4.23 d	0.50 f	69.90 b	9.57bc	139.30 e	128.60 e
**LSD 0.05**	**0.31**	**0.36**	**0.28**	**4.53**	**0.63**	**7.76**	**10.88**

S_1_ = 0% NaCl, S_2_ = 0.5% NaCl, S_3_ = 1% NaCl, S_4_ = 1.5% NaCl, Zn_1_ = 0 mg kg^-1^ Zn, Zn_2_ = 5 mg kg^-1^ Zn, Zn_3_ = 10 mg kg^-1^ Zn, Any two means followed by same letter within a column are statistically similar to each other (p > 0.05)

Plants grown under 0% salinity accumulated the highest amount of K, whereas plants form rest of the salinity levels accumulated similar amounts of K. Similarly, plants grown under Zn_5_ level acquired the highest amount of K, whereas Zn_0_ application resulted in the lowest K accumulation. In the same way, plants grown under 0% salinity with Zn_10_ acquired the highest amount of K, while similar amounts of K accumulated in the rest of interactions (Table 2).

The highest K/Na ratio was recorded under 0% salinity and Zn_10_ level. However, the lowest K/Na ratio was observed in 1 and 1.5% salinity levels and Zn_5_ and Zn_10_ levels. Regarding interactive effect, 0% salinity with Zn_10_ level had the highest K/Na ratio, whereas 1 and 1.5% salinity levels with all Zn application levels had the lowest K/Na ratio (Table 2).

The highest Zn uptake was noted for the plants grown under 0.5% salinity and Zn_10_ application, whereas Zn_0_ application and 1% salinity resulted in the lowest Zn accumulation. Regarding the interaction of salinity × Zn application levels, 0.5% salinity with Zn_10_ recorded the highest Zn accrual, while no salinity with no Zn application had the lowest Zn accumulation (Table 2).

Plants grown under 0.5% salinity and Zn_10_ application resulted in the highest Cu uptake, whereas Zn_0_ application and 1% salinity resulted in the lowest Cu accumulation. Regarding interactions, 0% salinity with Zn_5_ application recorded the highest Cu accrual, while 1% salinity with no Zn had the lowest Cu accumulation (Table 2).

The highest Mn uptake was noted for the plants grown under 0 and 0.5% salinity levels and Zn_0_, whereas Zn_10_ and 1.5% salinity resulted in the lowest Mn accumulation. Regarding interaction, no salinity with Zn_0_ application recorded the highest Mn accrual, while 1.5% salinity with all Zn levels had the lowest Mn accumulation (Table 2).

Plants grown under 1.5% salinity and Zn_0_ resulted in the highest Fe uptake, whereas 1% salinity and Zn_10_ resulted in the lowest Fe accrual. Regarding interaction, 1.5% salinity with Zn_0_ recorded the highest Fe accrual, while 0.5% salinity with Zn_10_ had the lowest Fe accumulation (Table 2).

**Fig 7:**
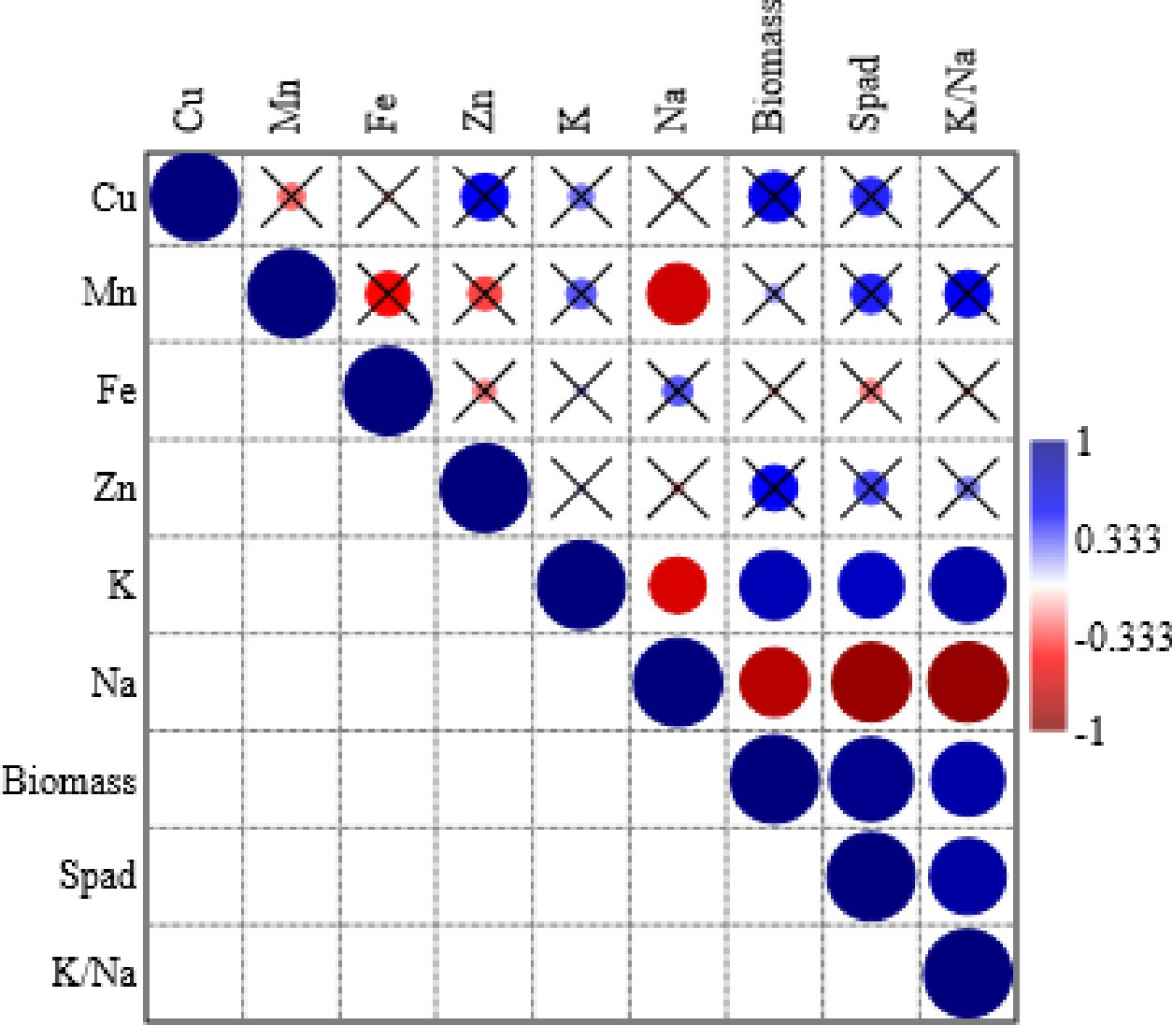
Correlation matrix of biomass and nutrient acquisition traits of Basil (*Ocimum basilicum* L.) grown under different NaCl salinity and Zn application levels. The size and color of the circles indicate the strength of correlation, whereas cross marks indicate that correlation is non-significant (p> 0.05).

Different growth and nutrient uptake traits had no positive correlation with each other; however, some traits were negatively correlated with each other (Fig 7). The Na accumulation had significant negative correlations with biomass production, chlorophyll index, K/Na ratio and accumulation of Mn and K (Fig 7). All other traits exhibited no positive/negative correlation.

## Discussion

Salinity significantly reduces plant growth depending on salt concentration and growth stage of the plants [6, 7]. Inhibition of photosynthesis is among the first indicators of salinity stress. Numerous studies have reported photosynthetic changes in crop plants in response to salinity stress [36–39]. Generally, photosynthesis is retarded by increasing salt levels [36, 37, 40, 41]. Stomatal and non-stomatal factors are responsible for retarded photosynthesis under salinity stress [42]. Salinity decreases CO_2_ assimilation and diffusion from the stomata to mesophyll cells [43] or alters photosynthetic mechanism [44].

Biomass production and chlorophyll index were reduced under increasing salinity levels in the current study. The decreased chlorophyll index and biomass production can be explained with ion toxicity caused by excessive salt levels. Results revealed that damage caused by salinity decreased with Zn application. The positive effect of Zn application on reduction of salt damage has been reported by several researchers [22–24, 45, 46]. Daneshbakhsh et al. [22] reported that negative effect of salt decreased with Zn application depending on genotype and salt concentration. In addition, K accumulation decreased under salinity; however, Zn application improved K uptake. Similarly, Na accumulation increased under salinity, while Zn application decreased Na concentration. Similar results have been reported in the current study. Results revealed significant decrease in biomass production with increasing salinity levels. However, basil managed to survive high salt stress. With increasing salinity levels, decreases in growth were higher in roots than in leaves [47]. The increased Na accumulation and a reduction in K, Zn, Cu and Mn concentrations could be responsible for decreased biomass. An earlier study has also reported that biomass of basil was decreased with increasing salinity [6, 7].

Nutrient uptake was significantly altered by different salinity levels included in the study. Decline in ion accumulation and selectivity has been well documented in wheat [48], sorghum [49], maize [50], barley [51] and rice [52]. However, basil exhibited a strong selectivity for K uptake, which improved K/Na ratio. Zinc reduces excessive Na uptake under saline environments by affecting structural integrity and permeability of stem cell membrane [23]. Zinc nutrition is effective in decreasing Na accumulation and improving K/Na ratio of plants under salinity. Therefore, cell membranes show high permeability or leakage of some compounds from the roots under Zn deficiency [25]. Zinc deficiency can lead to accumulation of toxic ions such as Na and Cl.

Different growth and nutrient uptake traits had no positive correlation with each other; however, some traits were negatively correlated (Fig 7). The Na accumulation had significant negative correlations with biomass production, chlorophyll index, K/Na ratio and accumulation of Mn and K (Fig 7). All other traits exhibited no positive/negative correlation. This indicated that Na accumulation has been the prime source of decreased growth and disturbed nutrient acquisition traits in the current study. This study suggested that field trials are necessary in saline and Zn deficit regions. In addition, Zn has potential to play a protective effect up to a certain extent in ameliorating salt damage.
